# Supplementary material for: Secretion and endocytosis in subapical cells support hyphal tip growth in the fungus Trichoderma reesei
Source: Nat Commun. 2025 May 12;16:4402. doi: 10.1038/s41467-025-59606-4 (PMC12069525; doi:10.1038/s41467-025-59606-4)
Supplement: Supplementary file 1 — Supplementary Information [file 41467_2025_59606_MOESM1_ESM.pdf]

Supplementary Information

Title: Secretion and endocytosis in subapical cells support hyphal tip growth in the fungus *Trichoderma reesei*

Authors: Schuster, M., Kilaru, S., Wösten, H. A. B., Steinberg, G.

Contents

Supplementary Tables
Supp. Table 1: Strains and their experimental usage.
Supp. Table 2: Plasmids used in this study.
Supp. Table 3: Hyphal "core growth units" in filamentous ascomycetes.
Supp. Table 4: Amino acid sequence analysis of predicted *T. reesei* proteins.
Supp. Table 5: Primers used in this study.

Supplementary Figures and Figure Legends
Supp. Figure 1: Organisation of the cloning vectors.
Supp. Figure 2: Re-establishment of a growing tip cell after laser injury of the 1st cell.
Supp. Figure 3: Response of *T. reesei* tip cells to laser injury of the 2nd cell.
Supp. Figure 4: Mitotic spindles in subapical cells and MT nucleation at septa.
Supp. Figure 5: Motility of a SVs in *T. reesei* hyphal cells.
Supp. Figure 6: Contrast-inverted kymograph of EE motility.
Supp. Figure 7: Analysis of anterograde and retrograde cross-septum EE motility.
Supp. Figure 8: The ER and the Golgi complex in apical and subapical hyphal cells.
Supp. Figure 9: Motility of fluorescent 1,3-β-glucan synthase and SVs and EEs.
Supp. Figure 10: Frequency of EE motility in Δ*Trhok1* mutants.

Supplementary Methods

Supplementary References

Supplementary Tables

Supplementary Table 1 Strains and their experimental usage

Name	Experimental usage	Reference
QM6a_TrmsGSso1	Fig.1a, 1e-i; Supp. Fig. 2; Supp. Fig. 3a, 3b; Supp. Movie 2	This study
QM6a	Fig. 1b; Fig. 9c, 9d	1
QM6a_TrmChSso1_Hex1TrmsG	Fig. 1c, 1d; Supp. Movie 1	This study
QM6a_TrmsGRab5_TrmChSso1	Fig. 1j, 1k; Fig. 2a-c; Fig. 5a-f; Fig. 9b; Supp. Fig. 6; Supp. Fig. 7; Supp. Fig. 10; Supp. Movie 4; Supp. Movie 9; Supp. Movie 10; Supp. Movie 11	This study
QM6a_TrmsG_TrmChSso1	Fig. 2d, 2e; Supp. Movie 3	This study
QM6a_ TrmChSso1_TrmsGTub1	Fig. 3a-e; Supp. Fig. 4a-c; Supp. Movie5	This study
QM6a_Lifeact-TreG	Fig. 3f, 3g; Fig. 6b-f; Supp. Movie 12	This study
QM6a_TrmsG$_2$Sec4_TrmChSso1	Fig. 4a-f; Supp. Fig. 5; Supp. Movie 6; Supp. Movie 7; Supp. Movie 8	This study
QM6a_ TrmChSso1	Fig. 6a	This study
QM6a_Cal^{s}TreG-HDEL	Fig. 7a; Supp. Fig. 8a	This study
QM6a _Ktr1TreG	Fig. 7b; Supp. Fig. 8b	This study
QM6a_ TrmChSso1_TrmsGSnc1	Fig. 7c,e,f,g; Supp. Movie 14	This study
QM6a_TrmsG$_2$Sec4_TrmChSnc1	Fig. 7d; Supp. Movie 13	This study
QM6a_TreGExo70	Fig. 7h	This study
QM6a_TrmCh$_2$Gcs1_TrmsGSso1	Fig. 8b, 8e-i	This study
QM6a_TrmsG$_2$Sec4_TrmCh$_2$Gcs1	Fig. 8c; Supp. Fig. 9a; Supp. Movie 15	This study
QM6a_TrmCh$_2$Gcs1_TrmsGRab5	Fig. 8d; Supp. Fig. 9b; Supp. Movie 16; Supp. Movie 17	This study
QM6a _TrmChSso1_paGGcs1	Fig. 8j, 8k	This study
QM6a_ΔHok1_TrmsGRab5	Fig. 9b; Supp. Fig. 10	This study
QM6a_ΔHok1	Fig. 9c, 9d	This study

TrmsG: *T. reesei* codon-optimised monomeric superfolder green fluorescent protein; Sso1: a syntaxin-like plasma membrane protein; TrmCh: *T. reesei* codon-optimised monomeric Cherry; Hex1: a major protein in Woronin bodies; Rab5: small endosomal GTPase; Tub1: α-tubulin; Lifeact : 17 residues from the actin binding protein ABP140p codon optimised for *T. reesei*; TreG: *T. reesei* codon optimised monomeric enhanced green fluorescent protein; Sec4: small GTPase; Cal^s: Signal sequence of calreticulin from rabbit; HDEL: endoplasmic reticulum retention signal; Ktr1: α-1,2-mannosyltransferase, Snc1: synaptobrevin-like vSNARE protein; Exo70: exocyst subunit; Gcs1: glucan synthase; paG: photo-activatable monomeric green fluorescent protein;. Δ: deletion; Hok1: Hook protein in *T. reesei*.

Supplementary Table 2 Plasmids used in this study

Name	Description	Reference
pTrCTrmsGFPSso1	P*tub1-trmsgfp-sso1*, *cbx*^{R}	This study
pTrHTrmCherrySso1	P*tub1-trmCherry-sso1*, *hyg*^{R}	2
pTrCHex1TrmsGFP	P*tub1-hex1-trmsgfp*, *cbx*^{R}	This study
pTrCTrmsGFPRab5	P*tub1-trmsgfp-rab5*, *cbx*^{R}	This study
pTrCTrmsGFP	P*tub1-trmsgfp*, *cbx*^{R}	3
pTrCTrmsGFPTub1	P*tub1-trmsgfp-tub1*, *cbx*^{R}	This study
pTrCLifeactTreGFP	P*tub1-lifeact-tregfp*, *cbx*^{R}	This study
pTrHTrmsGFP$_2$Sec4	P*sec4- trmsgfp*$_2$-sec4, *hyg*^{R}	This study
pTrCTrmCherrySso1	P*tub1-trmCherry-sso1*, *cbx*^{R}	This study
pTrCCal^{s}TreGFPHDEL	P*tub1-cal*^{s}*-tregfp-hdel*, *cbx*^{R}	This study
pTrHKtr1TreGFP	P*ktr1- ktr1- tregfp, hyg*^{R}	This study
pTrCTrmsGFPSnc1	P*snc1- trmsgfp-snc1, cbx*^{R}	This study
pTrCTrmCherrySnc1	P*snc1- trmCherry-snc1, cbx*^{R}	This study
pTrHTreGFPExo70	P*exo70-tregfp-exo70*, *hyg*^{R}	This study
pTrCTrmCherry$_2$Gcs1	P*gcs1- trmCherry*$_2$*-gcs1, cbx*^{R}	This study
pTrHTrmsGFPSso1	P*tub1-trmsgfp-sso1*, *hyg*^{R}	This study
pTrHTrmsGFPRab5	P*tub1-trmsgfp-rab5*, *hyg*^{R}	This study
pTrCpaGFPGcs1	P*gcs1- pagfp-gcs1, cbx*^{R}	This study
pTrHΔHok1	Δ*hok1, hyg*^{R}	This study

p: plasmid; C or cbx^R, carboxin resistance; TrmsGFP: *T. reesei* codon optimised monomeric superfolder green fluorescent protein; Sso1: a syntaxin-like plasma membrane protein; P: promoter; *tub1*: α-tubulin; H or hyg^R: hygromycin resistance; Hex1: a major protein in Woronin bodies; TrmCherry: *T. reesei* codon optimised monomeric Cherry; Rab5: small endosomal GTPase; Lifeact : 17 residues from the actin binding protein ABP140p codon optimised for *T. reesei*; TreGFP: *T. reesei* codon optimised monomeric enhanced green fluorescent protein; Sec4: small GTPase; Cal^s: Signal sequence of calreticulin from rabbit; HDEL: endoplasmic reticulum retention signal; Ktr1: α-1,2-mannosyltransferase, Snc1: synaptobrevin-like vSNARE protein; Exo70: exocyst subunit; Gcs1: glucan synthase; paG or *pagfp*: photo-activatable monomeric green fluorescent protein; Δ: deletion; Hok1: Hook protein in *T. reesei.*

Supplementary Table 3 Hyphal "core growth units" in filamentous ascomycetes

Name	Length (μm)	Width (μm)	Volume^c (μm^3)	CGU growth rate^d (μm h^{-1})	Hyphal growth rate^e (μm h^{-1})	Relative difference^f (%)	V/G ratio^g (μm$^{3/}$1 μm^{-1})	Reference
T. reesei	334^a	2.5^b	1,639.5	77 ± 66	191 ± 96	248.1 ↑$^{\&}$	21.2	this study
A. niger	363	5.7	9,262.9	112 ± 6	97 ± 5	13.4 ↓$^{\&}$	82.7	4
P. chrysogenum	243	4.5	3,864.7	80 ± 4	79 ± 4	1.25 ↓$^{\&}$	48.1	5

^{a} For dimension of 1st, 2nd and 3rd cell see Table 1 in main text.
^{b} For dimension of 1st, 2nd and 3rd cell see Table 1 in main text.
^{c} Estimated assuming a cylindrical shape ($V=\pi r^2 h$; r= radius of hypha, h= length of cell).
^{d} CGU= core growth unit, which comprises all cells that are required for hyphal extension.
^{e} Extension speed of un-wounded hyphae.
^{f} ↓↑ indicate the increase or decrease in extension rate in intact hyphae in percent of the CGU growth rate.
^{g} The volume of all cells in the core growth unit divided by its growth rate.

Supplementary Table 4 Amino acid sequence analysis of predicted *T. reesei* proteins

Name	Accession numbers*		Domains**	BLAST e-value	Identity/ similarity¶	Reference§
**Hex1**	*T. reesei* XP_006967461.1	*N. crassa* XP_963707.1	IF5A-like_N (3.3e-08) eIF5-a (1.4e-06)	9e-93	61.7/67.8	6
**Rab5**	*T. reesei* XP_006967529.1	*U. maydis* XP_011387349.1	Ras (3.6e-58)	6e-82	43.4/48.2	7
**Tub1**	*T. reesei* XP_006963464.1	*A. nidulans* XP_657920.1	Tubulin (1.7e-61) Tubulin_C (2.3e-50)	0.0	90.2/95.3	8
**Sec4**	*T. reesei* XP_006964176.1	*S. cerevisiae* EGA58852.1	Ras (2.3e-66)	1e-81	53.9/73.3	9
**Ktr1**	*T. reesei* XP_006962510.1	*S. cerevisiae* AJU11104.1	Glyco_transf_15 (1.9e-133)	4e-155	51.7/66.9	10
**Snc1**	*T. reesei* AAT78419.1	*A. nidulans* XP_682038.1	Synaptobrevin (1.6e-32)	9e-39	62.9/76.7	11,12
**Exo70**	*T. reesei* XP_006962255.1	*S. cerevisiae* NP_012450.1	Exo70 (3.4e-96)	5e-44	23.5/42.6	13
**Gcs1**	*T. reesei* XP_006965598.1	*U. maydis* XP_011387626.1	FKS1 (5.2e-35)	0.0	73.3/82.3	14
**Hok1**	*T. reesei* XP_006963103.1&	*U. maydis* XP_761698.1	None	9e-11	21.8/39.4	15

*NCBI data base at NCBI (https://www.ncbi.nlm.nih.gov/protein?cmd=retrieve)
**determined in PfamScan (https://www.ebi.ac.uk/jdispatcher/pfa/pfamscan) with error probability in brackets
$BLAST done at NCBI (https://blast.ncbi.nlm.nih.gov/Blast.cgi?PAGE=Proteins)
¶given as percentage of amino acid that are identical or similar to the reference sequence, as determined in EMBOSS Needle. (http://www.ebi.ac.uk/Tools/psa/emboss_needle/)
§reference for homologue used for comparison
&Annotation lacks the N-terminal 119 amino acids missing

Supplementary Table 5 Primers used in this study.

Name	Sequence (5′ to 3′)
SK-Tri-2	*GCAGTTGAGAATGGTGAGGCAGCGGTACAG*GCTCATGGAGTTCTCGAGCTTGC
SK-Tri-3	*CTGTACCGCTGCCTCACCATTCTCAACTGC*ACGCGGACCTGCCCCAAGGG
SK-Tri-7	*TTTTTTACCATTCGTACTCTCTGCGTGACT*TGGTCGAAGGCGAATGGACGCG
SK-Tri-10	*ACTGCTTGAAACACACACAACATGGATTCT*CGCCAGCGGAATCGCCTTCTTC
SK-Tri-11	ATGGTCAGCAAGGGCGAGGAGC
SK-Tri-18	*ATCCATGACTCCAACAGCAAAAGAAACAGC*CTAGTTCTTGTTTGTCAAAGCGAC
SK-Tri-19	GCTGTTTCTTTTGCTGTTGGAGTC
SK-Tri-26	CTTGTACAGCTCGTCCATGCCG
SK-Tri-33	*CTCCTCCTTGCTGATGCTCTCGAACTTCTTGATGAGGTCGGCGACGCCCAT*TGTGGACGATTTTGGAGTGTTTGATC
SK-Tri-34	*ATGGGCGTCGCCGACCTCATCAAGAAGTTCGAGAGCATCAGCAAGGAGGAG*ATGGTCAGCAAGGGCGAGGAGC
SK-Tri-45	TGGTCGAAGGCGAATGGACGCG
SK-Tri-49	*GGCGGCGGCCAGGCCGAGCAGGCCGAGCAGCAGCGGCACAGGGAGCAACAT*TGTGGACGATTTTGGAGTGTTTGATC
SK-Tri-50	*ATGTTGCTCCCTGTGCCGCTGCTGCTCGGCCTGCTCGGCCTGGCCGCCGCC*ATGGTCAGCAAGGGCGAGGAGC
SK-Tri-51	*AGCAAAAGAAACAGCTTACAGCTCGTCGTG*CTTGTAGAGCTCGTCCATGCCG
SK-Tri-52	*CACGACGAGCTGTAA*GCTGTTTCTTTTGCTGTTGGAGTC
SK-Tri-56	*ATCCATGACTCCAACAGCAAAAGAAACAGCTTA*GCCGCTGCTGCCGCTGCTGC
SK-Tri-69	CCGGGCTCAATCTTGCACTTGAC
SK-Tri-72	*ATCCATGACTCCAACAGCAAAAGAAACAGC*CTAGCAGGCACATCCATCCTTGG
SK-Tri-85	GAATTCGAGCTCGGTACCCAACT
SK-Tri-86	GCGTTAACACTAGTCAGATCTACC
SK-Tri-89	*TGGCAGGATATATTGTGGTGTAAACAAATT*ACCTCCGGTCGATCTGCAATCC
SK-Tri-91	*TCAGACAGTACATGCATGTTGCATGATGAT*TGGGTGGCCAATCGCTCAATGG
SK-Tri-94	*TAAACGCTCTTTTCTCTTAGGTTTACCCGC*GCAGCATCTCGGCTTCGTTCGC
SK-Tri-97	*CATGTTGTCCTCCTCGCCCTTGCTGACCAT*GATGAGAAAAAGTTACCTGAGAAAGG
SK-Tri-102	ACTGGTACAGTGGCGTGATGATG
SK-Tri-112	ATGGACAGCACCGAGAGCCTC
SK-Tri-127	ATGGTCAGCAAGGGCGAGGAGG
SK-Tri-129	CTTGTAGAGCTCGTCCATGCCG
SK-Tri-133	*TAAACGCTCTTTTCTCTTAGGTTTACCCGC*GCGTTAACACTAGTCAGATCTACC
SK-Tri-134	*TGGCAGGATATATTGTGGTGTAAACAAATT*ACTGTTGCTGGCAGGATCTGAAG
SK-Tri-135	*CAATATCAGTTGGGTACCGAGCTCGAATTC*TCCTCCGTTTAGTTCTGGTAGCC
SK-Tri-136	*CACCATGGTAGATCTGACTAGTGTTAACGC*GTGAAGCTTGATGCAGTTCATGCT
SK-Tri-137	*GCCGGTGAAGAGGCTCTCGGTGCTGTCCAT*GGCGGGCATCTTGGGTGAGAC
SK-Tri-139	*TAAACGCTCTTTTCTCTTAGGTTTACCCGC*AATTAGGATCTTGTTGACGCCCTC
SK-Tri-141	TTAACAGCACTTGCCGCTGGCG
SK-Tri-167	TGTACAAGATGGTCAGCAAGGGCGAGGAGG
SK-Tri-168	GCCCTTCTTGTACAGCTCGTCCATGCCGCC
SK-Tri-188	ATGGACAGCACCGAGAGCCTCTT
SK-Tri-189	TGTACAGCCGCTGCTGCCGCT
SK-Tri-191	*ATGGTTCGTCTCTCTCTCTCTACTGAATGC*GAAATTATTAATAGTAATTCCCGGCG
SK-Tri-192	GCATTCAGTAGAGAGAGAGAGACG
SK-Tri-193	CGAAACACTATCAGTCAACCACTC
SK-Tri-194	*AAGTTTGAGTGGTTGACTGATAGTGTTTCG*TGGGTGGCCAATCGCTCAATGG
SK-Tri-195	*AGCACCGGCGGCATGGACGAGCTGTACAAG*ATGTCGGGATATCCTGGCGGCG
SK-Tri-207	ATGGACAGCACCGAGAGCCTCT
SK-Tri-208	GCCGCTGCTGCCGCTGCTGC
SK-Tri-209	*ATCACCCACGGCAGCAGCGGCAGCAGCGGC*ATGGCGGAAGCTCCCAAGCCCA
SK-Tri-215	*TGGCAGGATATATTGTGGTGTAAACAAATT*TGCCGAACGCGCAGTGGAGTG
SK-Tri-216	*CAATATCAGTTGGGTACCGAGCTCGAATTC*TTCGCCCGACATATATGTAGCTCA
SK-Tri-217	*CACCATGGTAGATCTGACTAGTGTTAACGC*GATTCAAGGGGTTGGGCTTCATCT
SK-Tri-218	*TAAACGCTCTTTTCTCTTAGGTTTACCCGC*GAGGGCCTCGATGCCATGCTG
SK-Tri-219	GAATTCGAGCTCGGTACCCAACT
SK-Tri-220	GCGTTAACACTAGTCAGATCTACC
SK-Tri-221	GATCGCGTGTAAAGAGAGCGGAG
SK-Tri-222	CTAAGAAGACTCCTCTCGGAAGAG
SK-Tri-276	*ATCACCCACGGCAGCAGCGGCAGCAGCGGC*ATGCGTGAGGTTATCAGCATCAAC
SK-Tri-314	*TGGCAGGATATATTGTGGTGTAAACAAATT*CGAGAGAGCAACGCGTGCCGT
SK-Tri-315	*CAATATCAGTTGGGTACCGAGCTCGAATTC*GAGCCACCCGTGAACAGAGATC
SK-Tri-316	*CACCATGGTAGATCTGACTAGTGTTAACGC*GCCGGCTTCAGCTGCCGTGG
SK-Tri-317	*GGTGAAGAGCTCCTCGCCCTTGCTGACCAT*GACGCGTGCAGCGGCGAATCG
SK-Tri-318	*ATCACCCTCGGCATGGACGAGCTCTACAAG*ATGGCTGTCGGGGCGCTGTCC
SK-Tri-319	*TAAACGCTCTTTTCTCTTAGGTTTACCCGC*CATGTAGCAGTCGGTGGTAAGGTA
SK-Tri-326	CGAAGAATCCTTCAAGGTCTTTGAG
SK-Tri-327	CGCCGAGAGAGATCATGATGCTC
SK-Tri-330	CTTGTAGAGCTCGTCCATGCCGA
SK-Tri-365	*TGGCAGGATATATTGTGGTGTAAACAAATT*TACCGCCACATGTGCCGCTTCG
SK-Tri-366	*GGTGAAGAGCTCCTCGCCCTTGCTGACCAT*GTCTTGCTGGTTCTCCCAGCCT
SK-Tri-367	*CACCATGGTAGATCTGACTAGTGTTAACGC*GAGAAGTCGGGAGCTTTGACGAA
SK-Tri-368	*TAAACGCTCTTTTCTCTTAGGTTTACCCGC*GCAAGCAACCCTCAACCAAGTCC
SK-Tri-409	*CACGGCAGCAGCGGCAGCAGCGGCTGTACA*ATGCCTAGCAATCGCAACTACGAC
SK-Tri-412	*CTCAGATCAAACACTCCAAAATCGTCCACA*ATGGGTTACTACGACGACGAGGG
SK-Tri-413	*GCCGGTGAAGAGGCTCTCGGTGCTGTCCAT*CAGGCGAGAGCCGTGGACGAC
SK-Tri-416	*ATCACCCACGGCAGCAGCGGCAGCAGCGGC*ATGTCGAGCGGGCAGAATCCTTA
SK-Tri-437	GCATTCAGTAGAGAGAGAGAGACG
SK-Tri-438	CGAAACACTATCAGTCAACCACTC
SK-Tri-445	GATCCGCCAGGTCGAGGATCG
SK-Tri-453	GTACCATCCGTCTCCGACGGC
SK-Tri-454	GTGCCAACAGAAATCTGTGACTAAC
SK-Tri-455	AGTGCCGCTGTGTATTAATCGCC
SK-Tri-567	*TTGCCCTTTCTCAGGTAACTTTTTCTCATCC*ATGGTGAGCAAGGGCGAGGAGC
SK-Tri-568	*GCCGCCACCGCCGCCAGGATATCCCGACATATG*CTTGTACAGCTCGTCCATGCCG
SK-Tri-576	*TGGCAGGATATATTGTGGTGTAAACAAATT*AATGTCTCTCTTGCTGCATCACGC
SK-Tri-579	*GCCGGTGAAGAGGCTCTCGGTGCTGTCCAT*TTTGAGAGAATAAGGCGTTCAGAGG
SK-Tri-580	*CACGGCAGCAGCGGCAGCAGCGGCTGTACA*ATGGCCGACGCTCCGTACGATC
SK-Tri-581	*TAAACGCTCTTTTCTCTTAGGTTTACCCGC*TGGTAGCTCACCTGATGGAACGAT
SK-Tri-582	*ATGGTTCGTCTCTCTCTCTCTACTGAATGC*TGGGCTCATCGAAGGATCTCGG
SK-Tri-583	*AGTTTGAGTGGTTGACTGATAGTGTTTCGT*TGTTCCCGAACAGGTACAGTCAGA
SK-Tri-584	*CATGTTGTCCTCCTCGCCCTTGCTGACCAT*TTTGAGAGAATAAGGCGTTCAGAGG
SK-Tri-585	*AGCACCGGCGGCATGGACGAGCTCTACAAG*ATGGCCGACGCTCCGTACGATC
SK-Tri-586	GGCAGCCACACCACTGAGCAC
SK-Tri-587	ACTCCTCATGGCACAGAACCCTC

Italics indicates complementary gene sequence, allowing homologous recombination in *S. cerevisiae*.

Supplementary Figures and Figure Legends

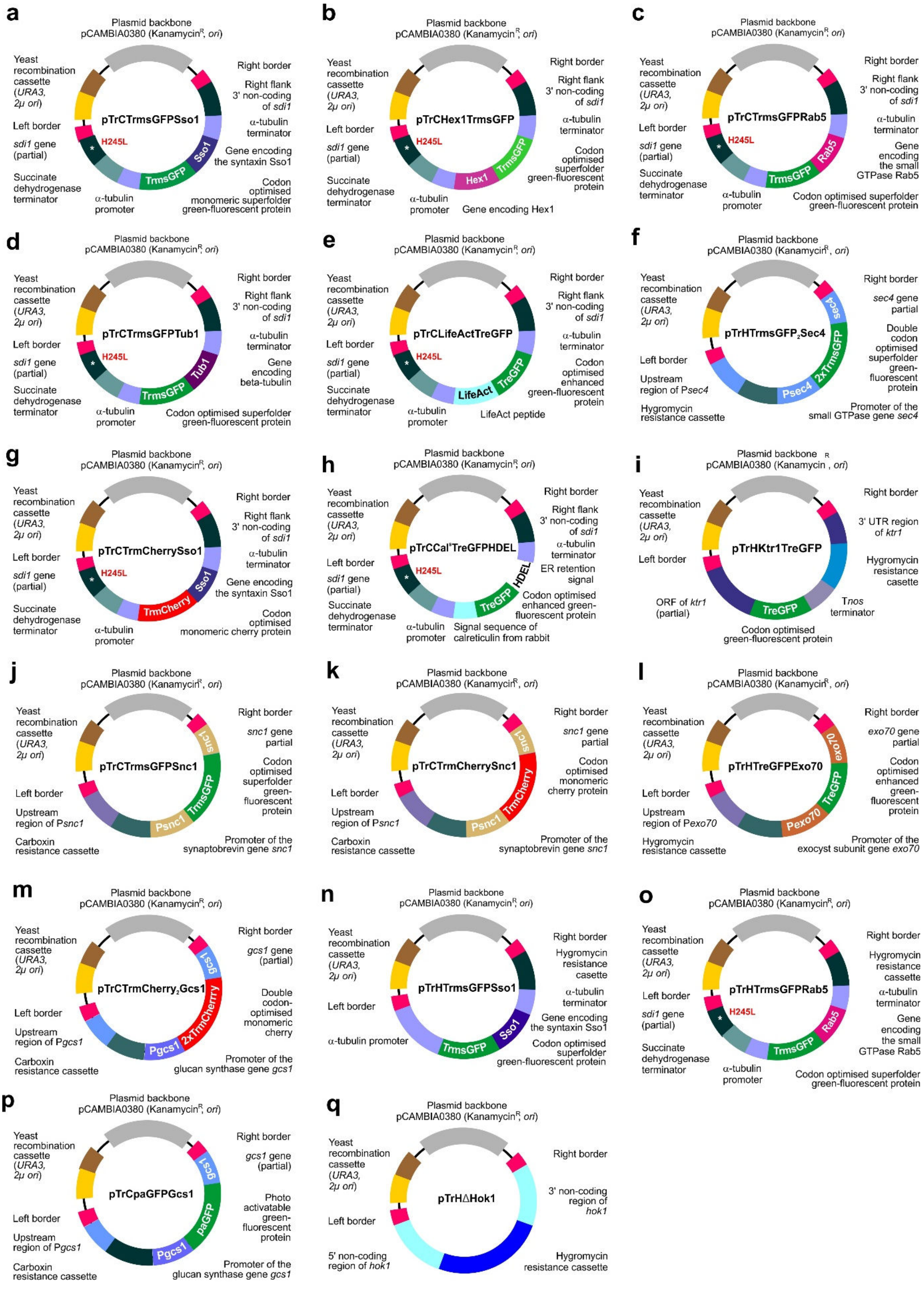

Supplementary Figure 1 Organisation of the cloning vectors.

a Plasmid pTrCTrmsGFPSso1 carries a fusion of codon-optimised superfolder *gfp* (TrmsGFP[3]) and a homologue of the plasma membrane syntaxin gene *sso1*[12].

b Plasmid pTrCHex1TrmsGFP expresses a fluorescent version of the Woronin body protein Hex1[6].

c Plasmid pTrCTrmsGFPRab5 introduces a fluorescent version of a homologue of the endocytic small GTPase Rab5, which localises to rapidly moving fungal EEs[7].

d Plasmid pTrCTrmsGFPTub1 carries a fusion of codon-optimised superfolder *gfp* (TrmsGFP[3]) and a homologue of α-tubulin encoding gene *tub1.*

e Plasmid pTrCLifeactTreGFP carries the 17 aa of the *S. cerevisiae* actin binding protein ABP140 encoding sequence[16], fused to the *T. reesei* codon-optimised enhanced *gfp* (TreGFP[3]).

f Plasmid pTrHTrmsGFP$_2$Sec4 carries a double *T. reesei* codon-optimised monomeric super-folder *gfp* (TrmsGFP) fused to the promoter and a part of the *sec4* gene, encoding a homologue of the small GTPase Sec4, which localises to SVs[9,17,18].

g Plasmid pTrCTrmCherrySso1 carries a fusion of codon-optimised *mCherry* (TrmCherry[3]) and a homologue of the plasma membrane syntaxin gene *sso1*[12].

h Plasmid pTrCCal^{s}TreGFPHDEL carries the 17 aa of the calreticulin from rabbit Cal^s and ER retention signal HDEL[19] fused to the *T. reesei* codon-optimised enhanced *gfp* (TreGFP[3]). Similar constructs successfully labelled the ER in *Ustilago maydis*[20] and *Zymoseptoria tritici*[21].

i Plasmid pTrHKtr1TreGFP carries a fusion of *ktr1* encoding a homologue of the α-1,2-mannosyltransferase Ktr1p in *S. cerevisiae*[10] and codon optimised enhanced *gfp* (TreGFP[3]).

j Plasmid pTrCTrmsGFPSnc1 carries a *T. reesei* codon-optimised superfolder *gfp* (TrmsGFP) fused to the promoter and a part of the *snc1* gene, encoding a synaptobrevin like V-SNARE SNC1, which localises to SVs[12].

k Plasmid pTrCTrmCherrySnc1 carries a *T. reesei* codon-optimised monomeric *Cherry* (TrmCherry) fused to the promoter and a part of the *snc1* gene, encoding a synaptobrevin like V-SNARE SNC1, which localises to SVs[12].

l Plasmid pTrHTreGFPExo70 carries a fusion of *exo70* encoding a homologue of the exocyst subunit Exo70 in *S. cerevisiae*[13] and codon optimised enhanced *gfp* (TreGFP[3]).

m Plasmid pTrCTrmCherry$_2$Gcs1 carries a double *T. reesei* codon-optimised *mCherry* (TrmCherry) fused to the part of the *gcs1* gene, encoding a homologue of the 1,3-β-glucan synthase Gcs1 in *U. maydis*, which localises to SVs[14].

n Plasmid pTrHTrmsGFPSso1 carries a fusion of codon-optimised superfolder *gfp* (TrmsGFP) and a homologue of the plasma membrane syntaxin gene *sso1*.

o Plasmid pTrHTrmsGFPRab5 introduces a fluorescent version of a homologue of the endocytic small GTPase Rab5, which localises to rapidly moving fungal EEs[7].

p Plasmid pTrCpaGFPGcs1 carries a photo-activatable *gfp* (paGFP[22]) fused to the part of the *gcs1* gene, encoding a putative 1,3-β-glucan glucan synthase.

q Plasmid pTrHΔHok1 carries the hygromycin resistance cassette, the promoter sequence and a downstream sequence of a homologue of the endosomal motor adapter *hok1*[15], which enables integration into the *hok1* locus, thereby deleting the *hok1* open reading frame.

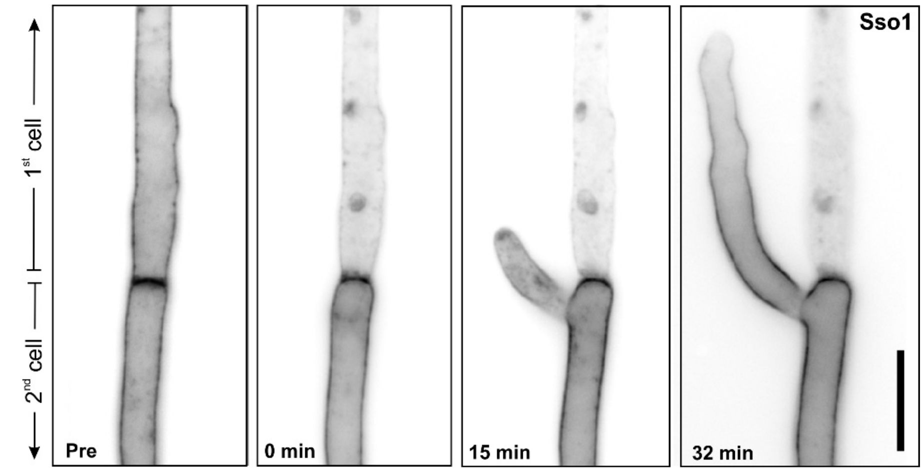

Supplementary Figure 2 Re-establishment of a growing tip cell after laser injury of the 1st cell (0 min). The tip cell was injured by a laser pulse. Within a few minutes, the 2nd cell establishes a new growth point and forms a growing tip cell. Images are contrast-inverted; time is given in minutes. Scale bar= 10 µm. Results shown in were confirmed in 2 independent experiments. All data are provided in the Source Data File.

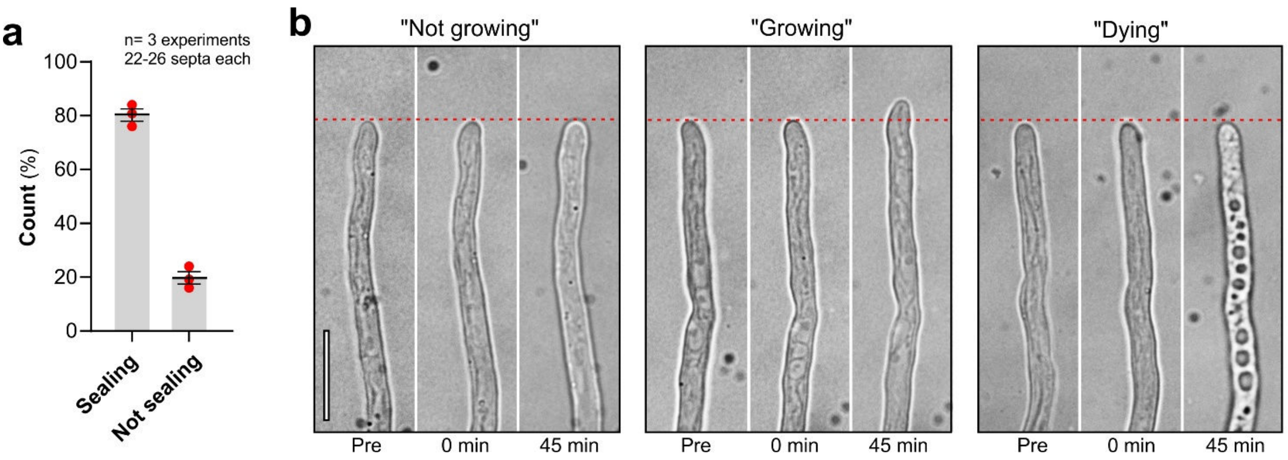

Supplementary Figure 3 Response of *T. reesei* tip cells to laser injury of the 2nd cell.

a Graph showing the number of hyphae that were sealing the first septum after injury of the 2nd cell. Sample size n= 3 independent experiments with 22-26 septa per experiment.

b Phenotype of tip cells before, shortly after and 45 minutes after injury of the 2nd cell. The 3 phenotypes are used in the main Fig. 1k. Scale bar= 10 µm.

Results shown in (**b**) were confirmed in 3 independent experiments. Bars in (**a**) represent mean ± SEM, average of individual experiments shown as red dots. All data are provided in the Source Data File.

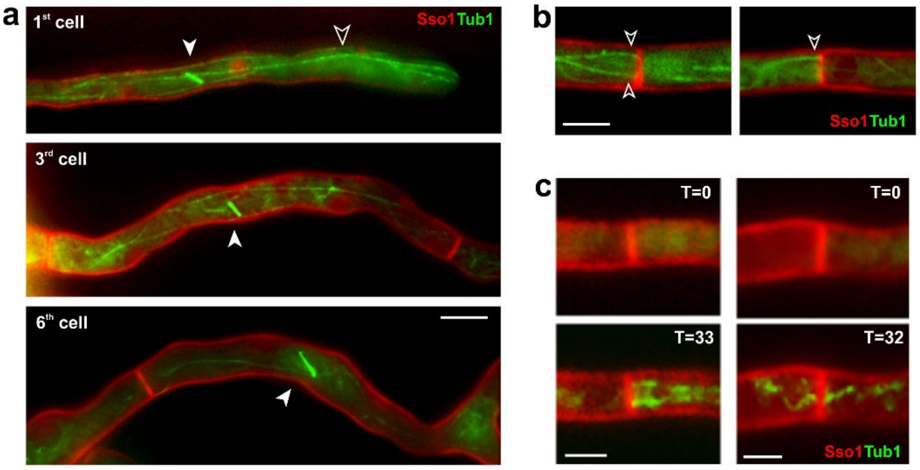

Supplementary Figure 4 Mitotic spindles in subapical cells and MT nucleation at septa.

a Mitotic spindles in hyphal cells of *T. reesei*. Note that the shown apical cell (1st cell) contains bundles of MTs (open arrowhead) and a spindle (closed arrowhead). Spindles are also present in subapical cells (3rd and 6th cell). Scale bar= 5 µm.

b MTs ending at septa. The tip of the hypha is located to the right. Scale bar= 3 µm.

c Reappearance of MTs at a septum after benomyl treatment and washout of the inhibitor. No MTs are found after 30 min incubation with 12 µM benomyl (T=0); faint MTs appear at the septum after extended time in fresh medium (T=32, T=33). This result suggests the existence of septal microtubule-organising centres. Scale bars= 2 µm.

Results shown in (**a**,**c**) were confirmed in 2 independent experiments, in (**b**) in 3 independent experiments. All data are provided in the Source Data File.

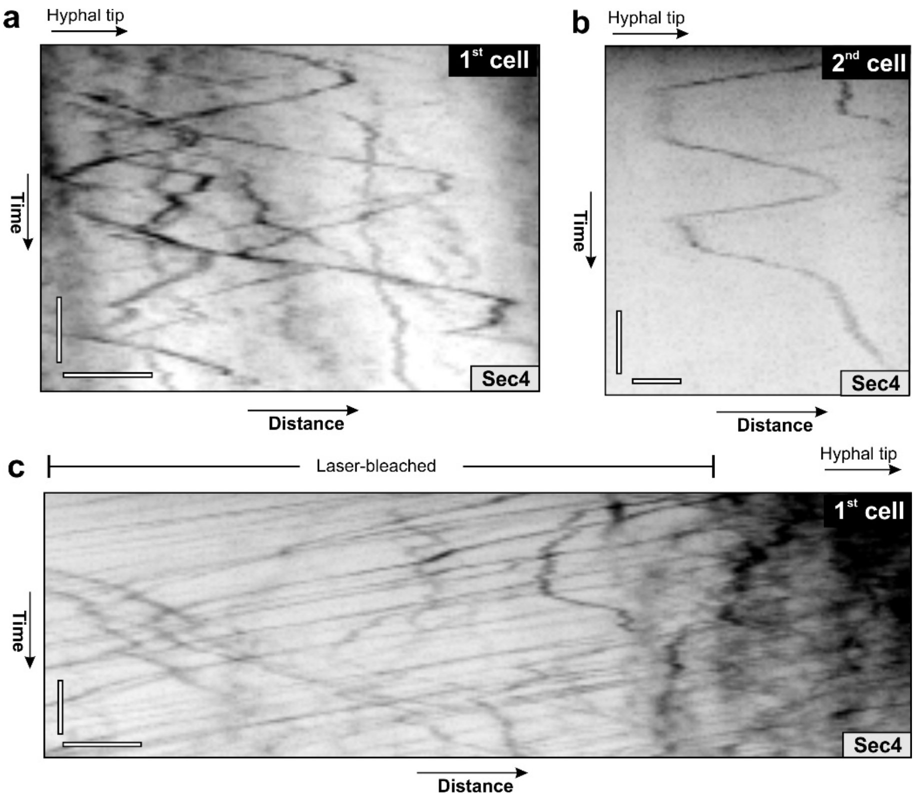

Supplementary Figure 5 Motility of a SVs in *T. reesei* hyphal cells.

a Bi-directional motility of SVs, labelled with TrmsGFP$_2$-Sec4, in the middle region of a 1st cell. Horizontal scale bar= 1 µm; vertical scale bar= 3 s.

b Bi-directional motility of SVs, labelled with TrmsGFP$_2$-Sec4, in a subapical 2nd cell.

Horizontal scale bar= 1 µm; vertical scale bar= 3 s.

c Retrograde motility of SVs, labelled with TrmsGFP$_2$-Sec4, at ~25-40 µm behind the hyphal tip; photo-bleached region is indicated above image. Horizontal scale bar= 1 µm; vertical scale bar= 3 s.

All kymographs are contrast-inverted; Results shown were confirmed in 3 independent experiments. Data are provided in the Source Data File.

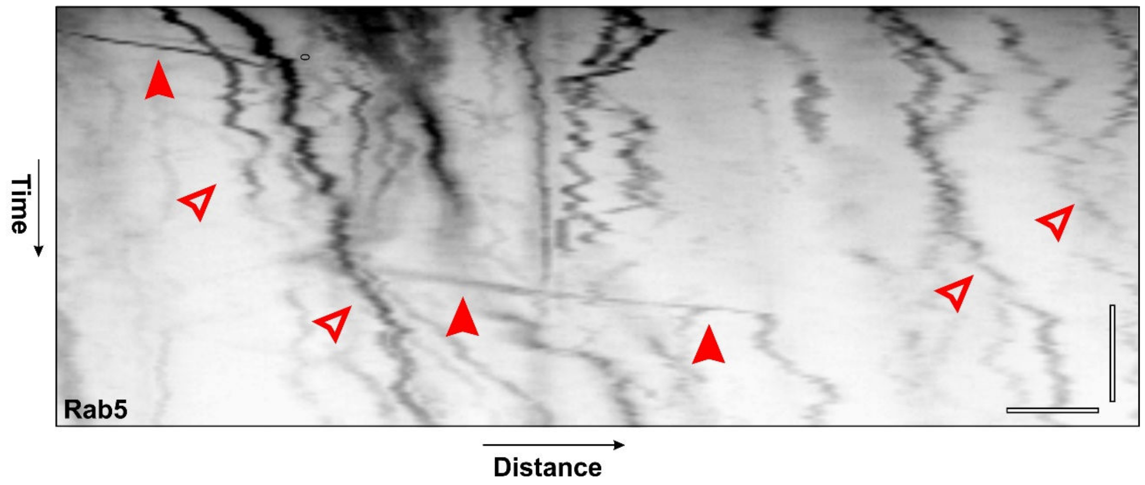

Supplementary Figure 6 Contrast-inverted kymograph of EE motility. TrmsGFP-Rab5-positive organelles show diffusional behaviour (open arrowheads) and rapid directed motility closed arrowhead. Horizontal scale bar= 2 µm, vertical scale bar= 5 s. Results shown confirmed in 3 independent experiments. Data were obtained from growing hyphae and are provided in the Source Data File.

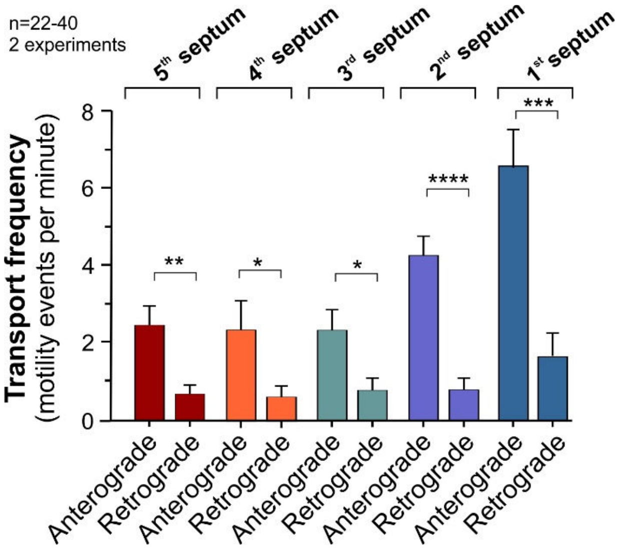

Supplementary Figure 7 Analysis of anterograde and retrograde cross-septum EE motility. Data are show as mean ± SEM. Statistical testing used Student's t-test with Welsh correction; *= two-tailed P value of 0.0235 (3rd septum) and 0.0446 (4th septum); **= two-tailed P values of 0.0035; ***= two-tailed P value of 0.0002; ****= two-tailed P values <0.0001. Sample size n= 22 septum (1st), n= 35 septum (2nd and 3rd), n= 34 septum (4th) and n= 37 septum (5th) from 2 independent experiments. All data were obtained from growing hyphae and are provided in the Source Data File.

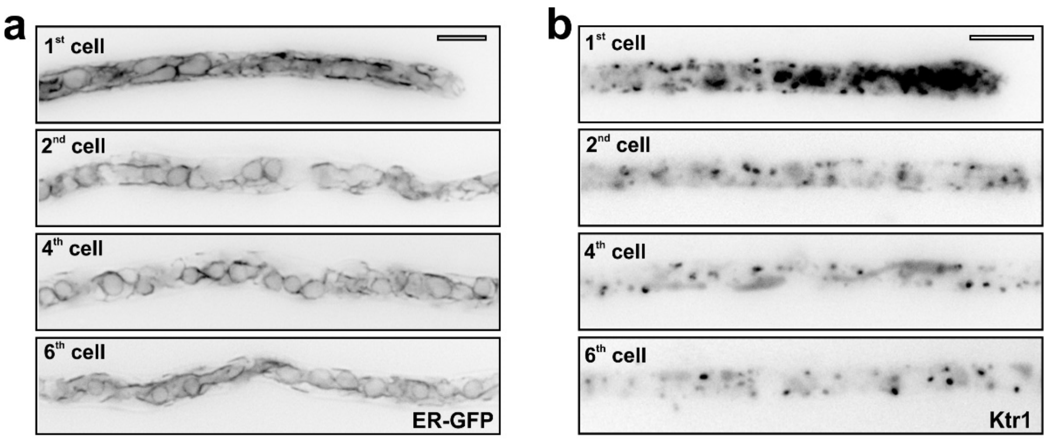

Supplementary Figure 8 The ER and the Golgi complex in apical and subapical hyphal cells.

a Contrast-inverted images of the endoplasmic reticulum marker ER-GFP (Cal^{s}-TreGFP-HDEL) in the 1st cell and in the 2nd, 4th and 6th cell. Scale bar= 5 µm.

b Contrast-inverted images of the Golgi marker TrKtr1-TreGFP localisation in the 1st cell and in the 2nd, 4th and 6th cell. Scale bar= 5 µm.

Results shown were confirmed independently in 2 experiments. All data are provided in the Source Data File.

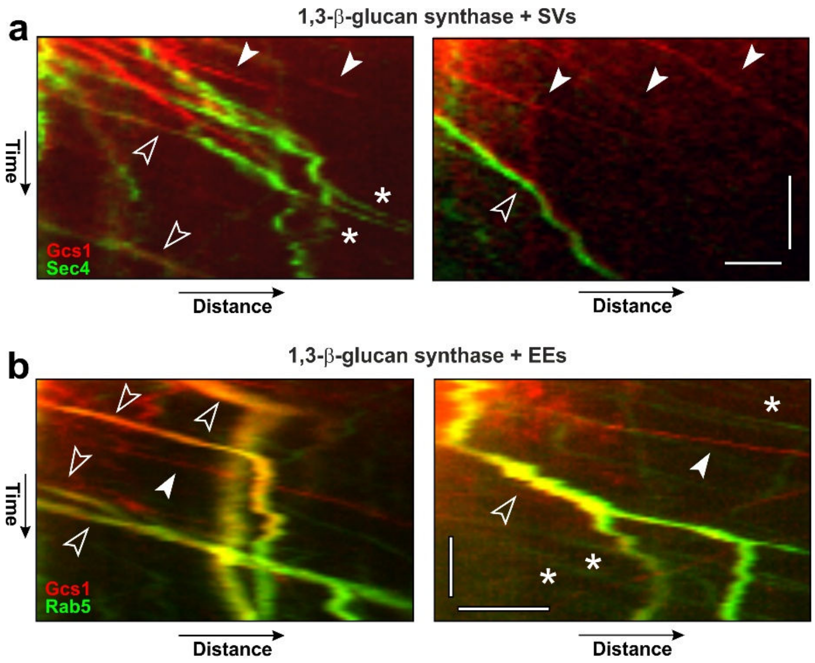

Supplementary Figure 9 Motility of fluorescent 1,3-β-glucan synthase and SVs and EEs.

a Kymographs showing SVs, labelled with TrmsGFP$_2$-Sec4 (green) and *T. reesei* 1,3-β-glucan synthase glucan synthase (TrmCherry$_2$-Gcs1; red) in a hyphal cell.

While some signal co-travel (open arrowhead), other TrmCherry2-Gcs1 do not co-localise with TrmsGFP2-Sec4, suggesting that these cell-wall synthases are not delivered to the apex by SVs. Open arrowheads: 1,3-β-glucan synthase in SVs (yellow); closed arrowheads: 1,3-β-glucan synthase travelling independent of SVs; asterisk: SVs travelling without 1,3-β-glucan synthase. Horizontal scale bar= 2 µm; vertical scale bar= 3 s. See also Supplementary Movie 12.

b Kymographs showing EEs, labelled with TrmsGFP-Rab5 (EEs; green) and 1,3-β-glucan synthase (TrmCherry2-Gcs1; red); open arrowheads: 1,3-β-glucan synthase in EEs (yellow); closed arrowheads: 1,3-β-glucan synthase travelling independent of EEs; asterisk: EEs travelling without 1,3-β-glucan synthase. Horizontal scale bar= 2 µm; vertical scale bar= 3 s. See also Supplementary Movie 15 and 16.
Results shown were confirmed in 3 independent experiments. All data are provided in the Source Data File.

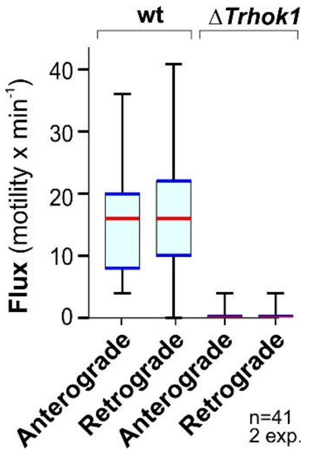

Supplementary Figure 10 Frequency of EE motility in Δ*Trhok1* mutants.

Quantitative analysis of bi-directional EE motility in wildtype strain QM6a (WT) and a *hok1* deletion mutant (Δ*hok1*). Data sets did not pass a normality test (Shapiro-Wilk test, P>0.05) and thus are given as Whiskers' plots (blue lines: 25/75 percentiles; red line: median; minimum and maximum at whiskers ends). Sample size n= 41 hyphae from 2 independent experiments. All data are provided in the Source Data File.

Supplementary Methods

pTrCTrmsGFPSso1. Plasmid pTrCTrmsGFPSso1 (Supplementary Fig. 1a) was obtained by *in vivo* yeast recombination of the following fragments: (i) a 12,849 bp fragment of plasmid pTrCTrmsGFP[3] (digested with *Bbv*CI and *Xba*I), (ii) a 714 bp *trmsgfp* (without stop codon; amplified with primers SK-Tri-207 and SK-Tri-208 from plasmid pTrCTrmsGFP) (See Supplementary Table 5 for all the primers), (iii) a 1245 bp *T. reesei* full-length *sso1* gene (with stop codon; amplified with primers SK-Tri-416 and SK-Tri-18 from QM6a genomic DNA), and (iv) a 1049 bp *T. reesei tub1* terminator (amplified with primers SK-Tri-19 and SK-Tri-10 from plasmid pTrCTrmsGFP).

pTrCHex1TrmsGFP. Plasmid pTrCHex1TrmsGFP (Supplementary Fig. 1b) was obtained by *in vivo* yeast recombination of the following fragments: (i) a 12,866 bp fragment of plasmid pTrCTrmCherry[3] (digested with *BstE*II), (ii) a 781 bp *T. reesei* full-length *hex1* gene (without stop codon; amplified with primers SK-Tri-412 and SK-Tri-413 from QM6a genomic DNA), (iii) a 717 bp *trmsgfp* (with stop codon; amplified with primers SK-Tri-112 and SK-Tri-56 from plasmid pTrCTrmsGFP), and (iv) a 1049 bp *T. reesei tub1* terminator (amplified with primers SK-Tri-19 and SK-Tri-10 from plasmid pTrCTrmsGFP).

pTrCTrmsGFPRab5. Plasmid pTrCTrmsGFPRab5 (Supplementary Fig. 1c) was obtained by *in vivo* yeast recombination of the following fragments: (i) a 12,849 bp fragment of pTrCTrmsGFP (digested with *Bbv*CI and *Xba*I), (ii) a 714 bp *trmsgfp* (without stop codon; amplified with primers SK-Tri-207 and SK-Tri-208 from plasmid pTrCTrmsGFP), (iii) a 825 bp *T. reesei* full-length *rab5* gene (with stop codon; amplified with primers SK-Tri-209 and SK-Tri-72 from QM6a genomic DNA), and (iv) a 1049 bp *T. reesei tub1* terminator (amplified with primers SK-Tri-19 and SK-Tri-10 from plasmid pTrCTrmsGFP).

pTrCTrmsGFPTub1. Plasmid pTrCTrmsGFPTub1 (Supplementary Fig. 1d) was obtained by *in vivo* yeast recombination of the following fragments: (i) a 12,849 bp fragment of pTrCTrmsGFP (digested with *Bbv*CI and *Xba*I), (ii) a 714 bp *trmsgfp* (without stop codon; amplified with primers SK-Tri-207 and SK-Tri-208 from plasmid pTrCTrmsGFP), (iii) a 1932 bp *T. reesei* full-length *tub1* gene with stop codon and a 1049 bp *T. reesei tub1* terminator (amplified as one fragment with primers SK-Tri-276 and SK-Tri-10 from QM6a genomic DNA).

pTrCLifeactTreGFP. Plasmid pTrCLifeactTreGFP (Supplementary Fig. 1e) was obtained by *in vivo* yeast recombination of the following fragments: (i) a 11,939 bp fragment of pTrCTreGFP (digested with *Bsr*GI), (ii) a 1018 bp *T. reesei tub1* promoter (amplified with primers SK-Tri-7 and SK-Tri-33 from plasmid pTrCTreGFP[3]), and (iii) a 720 bp *tregfp* (with stop codon) and 1049 bp *T. reesei tub1* terminator (amplified as one fragment with primers SK-Tri-34 and SK-Tri-10 from plasmid pTrCTreGFP). Note that chimeric primers SK-Tri-33 and SK-Tri-34 contain 17 amino acid lifeact sequences with *T. reesei*-codon optimised nucleotides.

pTrHTrmsGFP$_2$Sec4. Plasmid pTrHTrmsGFP$_2$Sec4 (Supplementary Fig. 1f) contains 2x *trmsgfp* fused to partial *T. reesei sec4* gene for targeted integration into the *sec4* locus of *T. reesei* using hygromycin as selection agent. To this end, first plasmid pTrHTrmsGFPSec4 was generated. Plasmid pTrHTrmsGFPSec4 was obtained by *in vivo* yeast recombination of the following fragments: (i) a 9958 bp fragment of pGΔWco1[23] (digested with *Xho*I), (ii) a 820 bp left flank covering the upstream of the *sec4* promoter sequence (amplified with SK-Tri-134 and SK-Tri-135 from QM6a genomic DNA), (iii) a 1510 bp of hygromycin resistance cassette (amplified with primers SK-Tri-85 and SK-Tri-86 from plasmid pCHyg-YR[24]); (iv) a 1000 bp of *sec4* promoter (amplified with SK-Tri-136 and SK-Tri-137 from QM6a genomic DNA), (v) a 720 bp *trmsgfp* (without stop codon; amplified with primers SK-Tri-188 and SK-Tri-189 from plasmid pTrCTrmsGFP), and (vi) a 800 bp 5' end of *sec4* gene (amplified with primers SK-Tri-409 and SK-Tri-139 from QM6a genomic DNA).

The single *trmsgfp* in plasmid pTrHTrmsGFPSec4 was replaced by 2x *trmsgfp* by conventional ligation method using T4 DNA ligase (New England Biolabs, Ipswich, UK). To this end, the 2x *trmsgfp* tag together with *Nco*I and *Bsr*GI restriction site sequences at the beginning and end of the 2x*trmsgfp*, respectively, was synthesised commercially (DNA 2.0, Menlo Park, CA, USA) resulting in pEX-K248-2xTrmsGFP. Plasmid pTrHTrmsGFP$_2$Sec4 (Supplementary Fig. 1f) was obtained by conventional ligation of the following fragments: (i) a 12,117 bp fragment of pTrHTrmsGFPSec4 (digested with *Bsr*GI and *Mlu*I), (ii) a 1002 bp fragment of pTrHTrmsGFPSec4 (digested with *Mlu*I and *Nco*I), and (iii) a 1,430 bp fragment of pEX-K248-2xTrmsGFP (digested with *Nco*I and *Bsr*GI).

pTrCTrmCherrySso1. Plasmid pTrCTrmCherrySso1 (Supplementary Fig. 1g) was obtained by *in vivo* yeast recombination of the following fragments: (i) a 12,849 bp fragment of plasmid pTrCTrmsGFP; (digested with *Bbv*CI and *Xba*I), and (ii) a 708

bp *trmCherry* without stop codon and a 1245 bp *T. reesei* full-length *sso1* gene with stop codon (amplified as one fragment with primers SK-Tri-69 and SK-Tri-18 from plasmid pTrHTrmCherrySso1).

pTrCCal^{s}TreGFPHDEL. Vector pTrCCal^{s}TreGFPHDEL (Supplementary Fig. 1h) was obtained by *in vivo* yeast recombination of the following fragments: (i) a 11,939 bp fragment of pTrCTreGFP (digested with *Bsr*GI), (ii) a 1018 bp *T. reesei tub1* promoter (amplified with primers SK-Tri-45 and SK-Tri-49 from plasmid pTrCTreGFP), (iii) a 51 bp Cal^{s}, 717 bp *tregfp* and 12 bp encoding HDEL (Cal^{s}TreGFPHDEL sequence; amplified with SK-Tri-50 and SK-Tri-51 from plasmid pTrCTreGFP) and (iv) a 1049 bp *T. reesei tub1* terminator (amplified with primers SK-Tri-52 and SK-Tri-10 from plasmid pTrCTreGFP). Note that chimeric primers SK-Tri-49 and SK-Tri-50 contain 17 amino acid sequence of calreticulin from rabbit (Cal^{s}), whilst the chimeric primers SK-Tri-51 and SK-Tri-52 contain 4 amino acid sequence of ER retention signal HDEL.

pTrHKtr1TreGFP. Plasmid pTrHKtr1TreGFP contains *tregfp* fused to partial *T. reesei ktr1* gene for targeted integration into the *ktr1* locus of *T. reesei* using hygromycin as selection agent (Supplementary Fig. 1i). It was obtained by *in vivo* yeast recombination of the following fragments: (i) a 9489 bp fragment of pTrHEB1TreGFP[3] (digested with *BamH*I and *Sal*I), (ii) a 2973 bp fragment of pTrHEB1TreGFP[3] (digested with *BamH*I and *Sal*I), (iii) a 814 bp of 3' end of *ktr1* gene (without stop codon; amplified with primers SK-Tri-365 and SK-Tri-366 QM6a genomic DNA), (iv) a 1510 bp hygromycin resistance cassette (amplified with primers SK-Tri-85 and SK-Tri-86 from plasmid pTrHEB1TreGFP), and (v) a 993 bp *ktr1* right flank covering the downstream of the *ktr1* gene, immediately after the stop codon (amplified with primers SK-Tri-367 and SK-Tri-368 QM6a genomic DNA).

pTrCTrmsGFPSnc1. Plasmid pTrCTrmsGFPSnc1 (Supplementary Fig. 1j) contains *trmsgfp* fused to partial *T. reesei snc1* gene for targeted integration into the *snc1* locus of *T. reesei* using carboxin as selection agent. It was obtained by *in vivo* yeast recombination of the following fragments: (i) a 9533 bp fragment plasmid pCZtGFP (digested with *BamH*I and *Hind*III), (ii) a 1000 bp left flank covering the upstream of the *snc1* promoter sequence (amplified with SK-Tri-576 and SK-Tri-582 from QM6a genomic DNA), (iii) a 3194 bp of carboxin resistance cassette (amplified with primers SK-Tri-437 and SK-Tri-438 from plasmid pTrCTrmCherry$_2$Gcs1 (this study); (iv) a 1050 bp of *snc1* promoter (amplified with SK-Tri-583 and SK-Tri-579 from QM6a genomic DNA), (v) a 720 bp *trmsgfp* (without stop codon; amplified with primers SK-

Tri-188 and SK-Tri-189 from plasmid pTrCTrmsGFP), and (vi) a 600 bp 5’ end of *snc1* gene (amplified with primers SK-Tri-580 and SK-Tri-581 from QM6a genomic DNA).

pTrCTrmCherrySnc1. Plasmid pTrCTrmCherrySnc1 (Supplementary Fig. 1k) contains *trmCherry* fused to partial *T. reesei snc1* gene for targeted integration into the *snc1* locus of *T. reesei* using carboxin as selection agent. It was obtained by *in vivo* yeast recombination of the following fragments: (i) a 9533 bp fragment plasmid pCZtGFP (digested with *BamH*I and *Hind*III), (ii) a 1000 bp left flank covering the upstream of the *snc1* promoter sequence (amplified with SK-Tri-576 and SK-Tri-582 from QM6a genomic DNA), (iii) a 3194 bp of carboxin resistance cassette (amplified with primers SK-Tri-437 and SK-Tri-438 from plasmid pTrCTrmCherry$_2$Gcs1 (this study); (iv) a 1050 bp of *snc1* promoter (amplified with SK-Tri-583 and SK-Tri-584 from QM6a genomic DNA), (v) a 708 bp *trmCherry* (without stop codon; amplified with primers SK-Tri-127and SK-Tri-129 from plasmid pStrataTrmCherry-*Bsr*GI), and (vi) a 600 bp 5’ end of *snc1* gene (amplified with primers SK-Tri-585 and SK-Tri-581 from QM6a genomic DNA).

pTrHTreGFPExo70. Plasmid pTrHTreGFPExo70 contains *tregfp* fused to partial *T. reesei exo70* gene for targeted integration into the *exo70* locus of *T. reesei* using hygromycin as selection agent (Supplementary Fig. 1l). It was obtained by *in vivo* yeast recombination of the following fragments: (i) a 9958 bp fragment of pGΔWco1[23] (digested with *Xho*I), (ii) a 1000 bp left flank covering the upstream of the *exo70* promoter sequence (amplified with SK-Tri-314 and SK-Tri-315 from QM6a genomic DNA), (iii) a 1510 bp of hygromycin resistance cassette (amplified with primers SK-Tri-85 and SK-Tri-86 from plasmid pCHyg-YR[24]); (iv) a 1000 bp of *exo70* promoter (amplified with SK-Tri-316 and SK-Tri-317 from QM6a genomic DNA), (v) a 720 bp *trmsgfp* (without stop codon; amplified with primers SK-Tri-11 and SK-Tri-330 from plasmid pTrCTrmsGFP), and (vi) a 1000 bp 5’ end of *exo70* gene (amplified with primers SK-Tri-318 and SK-Tri-319 from QM6a genomic DNA).

pTrCTrmCherry$_2$Gcs1. Plasmid pTrCTrmCherry$_2$Gcs1 (Supplementary Fig. 1m) contains 2x *trmCherry* fused to partial *T. reesei gcs1* gene for targeted integration into the *gcs1* locus of *T. reesei* using carboxin as selection agent. To this end, first plasmid pTrCTrmCherryGcs1 was generated. Plasmid pTrCTrmCherryGcs1 was obtained by *in vivo* yeast recombination of the following fragments: (i) a 9760 bp fragment of plasmid pCGen-YR[24] (digested with *Zra*I and *Xba*I), (ii) a 1010 bp left flank covering the upstream of the *gcs1* promoter sequence (amplified with primers

SK-Tri-89 and SK-Tri-191 from QM6a genomic DNA), (iii) a 3194 bp fragment of carboxin resistance cassette (amplified as two fragments with primers SK-Tri-192 and SK-Tri-2; SK-Tri-3 and SK-Tri-193 from QM6a genomic DNA), (iv) a 1010 bp of *gcs1* promoter (amplified with primers SK-Tri-194 and SK-Tri-97 from QM6a genomic DNA), (v) a 708 bp *trmCherry* (without stop codon; amplified with primers SK-Tri-127 and SK-Tri-26 from plasmid pStrataTrmCherry-*Bsr*GI), and (vi) a 1020 bp 5' end of *gcs1* gene (amplified with primers SK-Tri-195 and SK-Tri-94 from QM6a genomic DNA).

The single *trmCherry* in plasmid pTrCTrmCherryGcs1 was replaced by 2x *trmsgfp* by conventional ligation method using T4 DNA ligase (New England Biolabs, Ipswich, UK). To this end, first plasmid pStrataTrmCherry-*Bsr*GI was generated to introduce *Bsr*GI restriction site sequences at both ends of the *trmCherry* gene. Plasmid pStrataTrmCherry-*Bsr*GI was generated by ligating the PCR amplified product of full length 708 bp *trmCherry* gene (amplified with primers pTr-167 and pTr-168 from plasmid pTrCTrmCherry (note that the *Bsr*G1 restriction site sequences were added to these primers) into a StrataClone PCR cloning plasmid using StrataClone PCR Cloning Kit (Agilent Technologies, CA, USA). Finally**,** plasmid pTrCTrmCherryGcs1 was digested with *Bsr*GI and an additional copy of *trmCherry* was introduced as *Bsr*GI fragment (obtained from plasmid pStrataTrmCherry-*Bsr*GI by digestion with *Bsr*GI) by using T4 DNA ligase (New England Biolabs, Ipswich, UK) resulting in plasmid pTrCTrmCherry$_2$Gcs1 (Supplementary Fig. 1m).

pTrHTrmsGFPSso1. Plasmid pTrHTrmsGFPSso1 (Supplementary Fig. 1n) was obtained by *in vivo* yeast recombination of the following fragments: (i) a 12,785 bp fragment of plasmid pTrCTrmsGFPRab5 (this study (digested with *Ahd*I), (ii) a 1245 bp *T. reesei* full-length *sso1* gene (with stop codon; amplified with primers SK-Tri-416 and SK-Tri-18 from QM6a genomic DNA), and (iii) a 1049 bp *T. reesei tub1* terminator and a 1510 bp hygromycin resistance cassette (amplified as one fragment with primers SK-Tri-19 and SK-Tri-133 from plasmid pTrHTrmCherrySso1[3]).

pTrHTrmsGFPRab5. Plasmid pTrHTrmsGFPRab5 (Supplementary Fig. 1o) was obtained by *in vivo* yeast recombination of the following fragments: (i) a 12,785 bp fragment of pTrCTrmsGFPRab5 (this study (digested with *Ahd*I), (ii) a 825 bp *T. reesei* full-length *rab5* gene (with stop codon; amplified with primers SK-Tri-209 and SK-Tri-72 from QM6a genomic DNA), and (iii) a 1049 bp *T. reesei tub1* terminator and a 1510 bp hygromycin resistance cassette (amplified as one fragment with primers SK-Tri-19 and SK-Tri-133 from plasmid pTrHTrmCherrySso1.

pTrCpaGFPGcs1. Plasmid pTrCpaGFPGcs1 contains *pagfp* fused to partial *T. reesei gcs1* gene for targeted integration into the *gcs1* locus of *T. reesei* using carboxin as selection agent (Supplementary Fig. 1p). Plasmid pTrCpaGFPGcs1 was obtained by *in vivo* yeast recombination of the following fragments: (i) a 14,707 bp fragment of pTrCTrmCherry$_2$Gcs1 (this study (digested with *Pml*I and *BsrG*I), (ii) a 1010 bp of *gcs1* promoter (amplified with SK-Tri-194 and SK-Tri-97 from plasmid pTrCTrmCherry$_2$Gcs1), and (iii) a 717 bp *pagfp* (without stop codon; amplified with primers SK-Tri-567 and SK-Tri-568 from plasmid ppaGFP$_3$Dyn2[25]).

pTrHΔHok1. Plasmid pTrHΔHok1 (Supplementary Fig. 1q) was obtained by *in vivo* yeast recombination of the following fragments: (i) a 9760 bp fragment of pCGEN-YR[24] (digested with *Xba*I and *Zra*I), (ii) a 828 bp *T. reesei hok1* promoter (amplified with primers SK-Tri-215 and SK-Tri-216 from QM6a genomic DNA), (iii) a 1510 bp hygromycin resistance cassette (amplified with primers SK-Tri-219 and SK-Tri-220 from plasmid pCHyg-YR), and (iv) a 856 bp of the non-coding 3′ region of *hok1* (amplified with primers SK-Tri-217 and SK-Tri-218 from QM6a genomic DNA).

Supplementary References

1 Reese, E. T. History of the cellulase program at the U.S. army Natick Development Center. *Biotechnol. Bioeng. Symp.*, 9-20 (1976).

2 Kilaru, S., Schuster, M., Murray, R. & Steinberg, G. Optimal timing for Agrobacterium-mediated DNA transformation of *Trichoderma reesei* conidia revealed by live cell imaging. *Fungal Genet. Biol.* **142**, 103448 (2020). doi: 10.1016/j.fgb.2020.103448.

3 Kilaru, S., Schuster, M., Cannon, S. & Steinberg, G. Optimised red- and green-fluorescent proteins for live cell imaging in the industrial enzyme-producing fungus *Trichoderma reesei*. *Fungal Genet. Biol.* **138**, 103366 (2020). doi: 10.1016/j.fgb.2020.103366.

4 Tegelaar, M. & Wösten, H. A. B. Functional distinction of hyphal compartments. *Sci Rep* **7**, 6039 (2017). doi: 10.1038/s41598-017-06422-6.

5 Tegelaar, M., van der Lans, G. P. A. & Wösten, H. A. B. Apical but not sub-apical hyphal compartments are self-sustaining in growth. *Antonie Van Leeuwenhoek* **113**, 697-706 (2020). doi: 10.1007/s10482-020-01383-9.

6 Tenney, K. *et al.* Hex-1, a gene unique to filamentous fungi, encodes the major protein of the Woronin body and functions as a plug for septal pores. *Fungal Genet. Biol.* **31**, 205-217 (2000). doi: 10.1006/fgbi.2000.1230.

7 Fuchs, U., Hause, G., Schuchardt, I. & Steinberg, G. Endocytosis is essential for pathogenic development in the corn smut fungus *Ustilago maydis*. *Plant Cell* **18**, 2066-2081 (2006). doi: 10.1105/tpc.105.039388.

8 Morris, N. R., Lai, M. H. & Oakley, C. E. Identification of a gene for alpha-tubulin in *Aspergillus nidulans Cell* **16**, 437-442 (1979). doi: 10.1016/0092-8674(79)90019-9.

9 Guo, W., Roth, D., Walch-Solimena, C. & Novick, P. The exocyst is an effector for Sec4p, targeting secretory vesicles to sites of exocytosis. *EMBO J.* **18**, 1071-1080 (1999). doi: 10.1093/emboj/18.4.1071.

10 Lussier, M., Sdicu, A. M., Bussereau, F., Jacquet, M. & Bussey, H. The Ktr1p, Ktr3p, and Kre2p/Mnt1p mannosyltransferases participate in the elaboration of yeast O- and N-linked carbohydrate chains. *J. Biol. Chem.* **272**, 15527-15531 (1997). doi: 10.1074/jbc.272.24.15527.

11 Taheri-Talesh, N. *et al.* The tip growth apparatus of *Aspergillus nidulans*. *Mol Biol Cell* **19**, 1439-1449 (2008). doi: 10.1091/mbc.e07-05-0464.

12 Valkonen, M. *et al.* Spatially segregated SNARE protein interactions in living fungal cells. *J. Biol. Chem.* **282**, 22775-22785 (2007). doi: 10.1074/jbc.M700916200.

13 TerBush, D. R., Maurice, T., Roth, D. & Novick, P. The Exocyst is a multiprotein complex required for exocytosis in *Saccharomyces cerevisiae*. EMBO J. 15, 6483-6494 (1996). doi: 10.1002/j.1460-2075.1993.tb06093.x.

14 Schuster, M. *et al.* Co-delivery of cell-wall-forming enzymes in the same vesicle for coordinated fungal cell wall formation. *Nat. Microbiol.* **1**, 16149 (2016). doi: 10.1038/nmicrobiol.2016.149.

15 Bielska, E. *et al.* Hook is an adapter that coordinates kinesin-3 and dynein cargo attachment on early endosomes. *J. Cell Biol.* **204**, 989-1007 (2014). doi: 10.1083/jcb.201309022.

16 Riedl, J. *et al.* Lifeact: a versatile marker to visualize F-actin. *Nat. Methods* **5**, 605-607 (2008). doi: 10.1038/nmeth.1220.

17 Goud, B., Salminen, A., Walworth, N. C. & Novick, P. J. A GTP-binding protein required for secretion rapidly associates with secretory vesicles and the plasma membrane in yeast. *Cell* **53**, 753-768 (1988). doi: 10.1016/0092-8674(88)90093-1.

18 Jones, L. A. & Sudbery, P. E. Spitzenkörper, exocyst, and polarisome components in Candida albicans hyphae show different patterns of localization and have distinct dynamic properties. *Eukaryot. Cell* **9**, 1455-1465 (2010). doi: 10.1128/EC.00109-10.

19 Pelham, H. R. The retention signal for soluble proteins of the endoplasmic reticulum. *Trends Biochem. Sci.* **15**, 483-486 (1990). doi: 10.1016/0968-0004(90)90303-s.

20 Wedlich-Söldner, R., Schulz, I., Straube, A. & Steinberg, G. Dynein supports motility of endoplasmic reticulum in the fungus *Ustilago maydis*. Mol. Biol. Cell 13, 965-977 (2002). doi: 10.1091/mbc.01-10-0475.

21 Kilaru, S., Schuster, M., Ma, W. & Steinberg, G.. Fluorescent markers of various organelles in the wheat pathogen *Zymoseptoria tritici*. Fungal Genet. Biol. 105, 16-27 (2017). doi: 10.1016/j.fgb.2017.05.001.

22 Patterson, G. H. & Lippincott-Schwartz, J. A photoactivatable GFP for selective photolabeling of proteins and cells. *Science* **297**, 1873-1877 (2002). doi: 10.1126/science.1074952.

23 Kilaru, S. *et al.* *Zymoseptoria tritici* white-collar complex integrates light, temperature and plant cues to initiate dimorphism and pathogenesis. *Nat. Commun.* **13**, 5625 (2022). doi: 10.1038/s41467-022-33183-2.

24 Kilaru, S. & Steinberg, G. Yeast recombination-based cloning as an efficient way of constructing vectors for *Zymoseptoria tritici*. *Fungal Genet. Biol.* **79**, 76-83 (2015). doi: 10.1016/j.fgb.2015.03.017.

25 Schuster, M., Lipowsky, R., Assmann, M. A., Lenz, P. & Steinberg, G. Transient binding of dynein controls bidirectional long-range motility of early endosomes. *Proc. Natl. Acad. Sci. U S A*, 3618-3623 (2011). doi: 10.1073/pnas.1015839108.
