# Supplementary material for: Secretion and endocytosis in subapical cells support hyphal tip growth in the fungus Trichoderma reesei
Source: Nat Commun. 2025 May 12;16:4402. doi: 10.1038/s41467-025-59606-4 (PMC12069525; doi:10.1038/s41467-025-59606-4)
Supplement: Supplementary file 2 — Description of Additional Supplementary Files [file 41467_2025_59606_MOESM2_ESM.pdf]

Description of Additional Supplementary Files:

Supplementary Movie 1: Woronin bodies guarding a septal pore in *T. reesei*. Red: TrmCherry-Sso1 in the plasma membrane; green: Hex1- TrmsGFP in Woronin bodies. Scale bar= 2 µm.

Supplementary Movie 2: Laser dissection of a tip cell of *T. reesei*. Time after laser injury is given in seconds and milliseconds. The plasma membrane is labelled TrmsGFP-Sso1. Scale bar= 10 µm.

Supplementary Movie 3: Loss of cytoplasm from 1st cell after laser dissection of 2nd cell. The 1st cell is to the right; note that internal pressure bends the septum towards the wounded 2 nd cell and that cytoplasm leaks through the septum for several seconds. Time is given in seconds and milliseconds. Scale bar= 3 µm.

Supplementary Movie 4: Tip-ward intercellular cytoplasmic streaming in the 2nd and 3rd cell. EEs are labelled with TrmsGFP-Rab5; The plasma membrane is labelled with TrmCherry-Sso1; the 3rd cell is to the right; time is given in seconds and milliseconds. Scale bar= 5 µm.

Supplementary Movie 5: Microtubules running through a septum in *T. reesei*. Microtubules are visualised by fluorescent α-tubulin (TrmsGFP-Tub1), the plasma membrane is visualised by the fluorescent syntaxin TrmCherry-Sso1. Scale bar= 2 µm.

Supplementary Movie 6: Motility of SVs in the apical region of a hypha. SVs were visualised using fluorescent Sec4 GTPase (TrmsGFP2-Sec4). The laser-bleached area is indicated by a yellow box. Time is given in seconds and milliseconds. Scale bar= 10 µm.

Supplementary Movie 7: Importance of the cytoskeleton for motility of SVs. SVs were visualised using fluorescent Sec4 GTPase (TrmsGFP2-Sec4). Cells were incubated for 30 minutes in 10 µM benomyl and 10 µM latrunculin A; control experiments used equal amounts of DMSO. Time is given in seconds and milliseconds. Scale bar= 3 µm.

Supplementary Movie 8: Motility of SVs through the 1st septum. SVs were visualised using fluorescent Sec4 GTPase (TrmsGFP2-Sec4), the plasma membrane is visualised using TrmCherrySso1. Time is given in seconds and milliseconds. Scale bar= 2 µm.

Supplementary Movie 9: Motility of EEs in the sub-apical 4th cell. EEs were visualised using fluorescent Rab5 GTPase (TrmsGFP-Rab5). Time is given in seconds and milliseconds. Scale bar= 10 μm.

Supplementary Movie 10: Importance of the cytoskeleton for motility of EEs. Organelles were visualised using fluorescent Rab5 GTPase (TrmsGFPRab5). Cells were incubated for 30 minutes in 10 μM benomyl and 10 μM latrunculin A; control experiments used equal amounts of DMSO. Time is given in seconds and milliseconds. Scale bar= 3 μm.

Supplementary Movie 11: Directed motility of EEs through a septum. EEs were visualised using fluorescent Rab5 GTPase (TrmsGFP-Rab5), the plasma membrane is visualised using TrmCherrySso1. Time is given in seconds and milliseconds. Scale bar= 2 μm.

Supplementary Movie 12: Actin patch dynamics in sub-apical cells. Actin patches are visualised by a fusion of the Lifeact peptide (17 aa of the *S. cerevisiae* actin binding protein ABP140), was optimised to the codon usage of *T. reesei* and fused to a codon-optimised eGFP (Lifeact-TreGFP). Image series represents a top view. Time is given in seconds and milliseconds. Scale bar= 5 μm.

Supplementary Movie 13: Motility of TrmCherry-Snc1 and TrmsGFP2-Sec4 in a 2nd cell. Most signals co-localise, confirming that the v-SNARE travels in SVs. Circle indicates TrmCherry-Snc1 that is not travelling in SVs. Time is given in seconds and milliseconds. Scale bar= 1 μm.

Supplementary Movie 14: Delivery, tethering and fusion of a vesicle in a subapical 2nd cell. A TrmsGFPSnc1- carrying vesicle is delivered to the site of exocytosis. Arrowhead indicates pausing under the plasma membrane, prior to exocytosis and disappearance of the fluorescent signal. The plasma membrane is labelled with TrmCherrySso1. Time is given in seconds and milliseconds. Scale bar= 2 μm.

Supplementary Movie 15: Covisualisation of 1,3- β -glucan synthase and SVs. Motility of the cell wall synthase-containing carrier was visualised using TrmCherry2-Gcs1, SVs are visualised using TrmsGFP2-Sec4. Note that 1,3-β-glucan synthase cotravels with SVs (open arrowhead), but also moves independently of TrmsGFP2- Sec4 (closed arrowhead). Time is given in seconds and milliseconds. Scale bar= 2 μm.

Supplementary Movie 16: Covisualisation of 1,3- β -glucan synthase and EEs. Motility of the cell wall synthase-containing carrier was visualised using TrmCherry2-Gcs1, endosomes are

visualised using TrmsGFP-Rab5. Note that most β-glucan synthase co-travels with endosomes. Time is given in seconds and milliseconds. Scale bar= 2 μm.

Supplementary Movie 17: Co-travelling β-glucan synthase and EEs across a septum. Motility of the cell wall synthasecontaining carrier was visualised using TrmCherry2-Gcs1, endosomes are visualised using TrmsGFP-Rab5. Time is given in seconds and milliseconds; the septum is visualised in the right bright field image (red arrowheads). Scale bar= 3 μm.
