# Supplementary figures and images for: Secretion and endocytosis in subapical cells support hyphal tip growth in the fungus Trichoderma reesei
Source: Nat Commun. 2025 May 12;16:4402. doi: 10.1038/s41467-025-59606-4 (PMC12069525; doi:10.1038/s41467-025-59606-4)

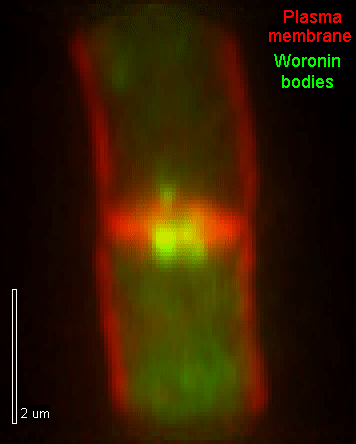

Supplement: Supplementary file 3 — Supplementary Movie 1 [file 41467_2025_59606_MOESM3_ESM.gif]

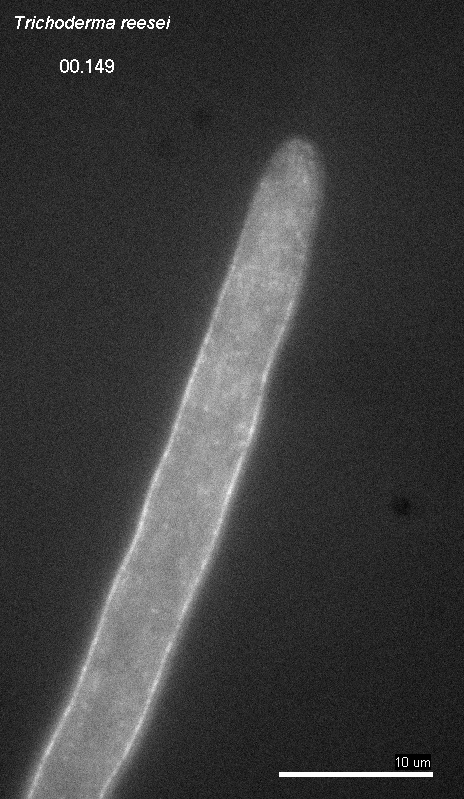

Supplement: Supplementary file 4 — Supplementary Movie 2 [file 41467_2025_59606_MOESM4_ESM.gif]

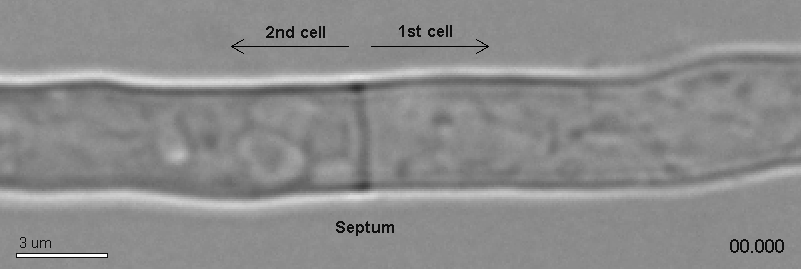

Supplement: Supplementary file 5 — Supplementary Movie 3 [file 41467_2025_59606_MOESM5_ESM.gif]

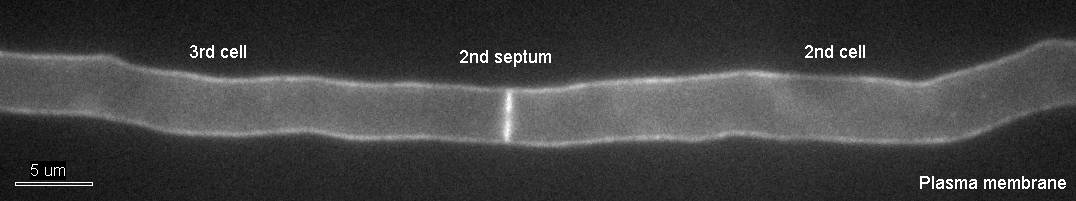

Supplement: Supplementary file 6 — Supplementary Movie 4 [file 41467_2025_59606_MOESM6_ESM.gif]

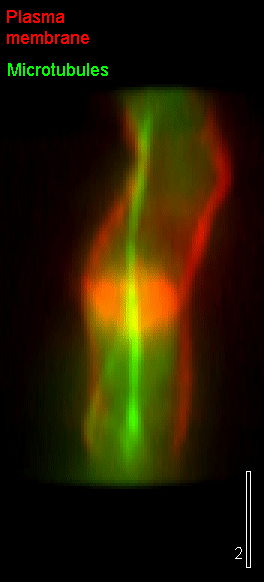

Supplement: Supplementary file 7 — Supplementary Movie 5 [file 41467_2025_59606_MOESM7_ESM.gif]

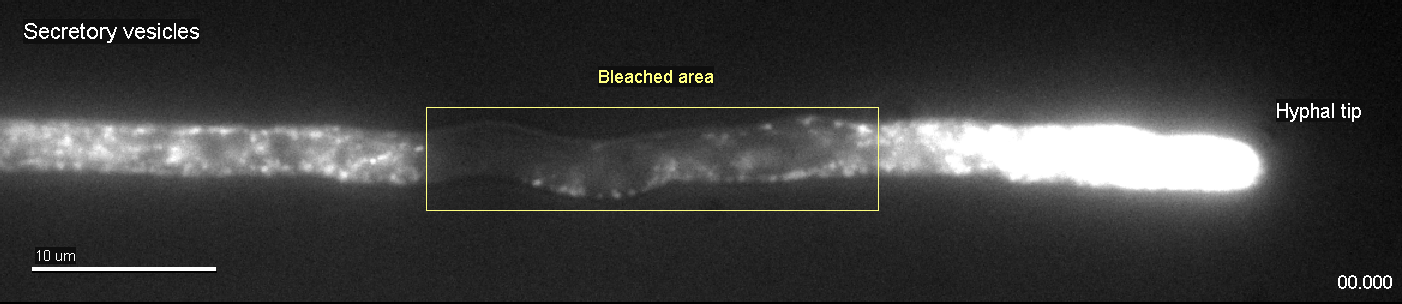

Supplement: Supplementary file 8 — Supplementary Movie 6 [file 41467_2025_59606_MOESM8_ESM.gif]

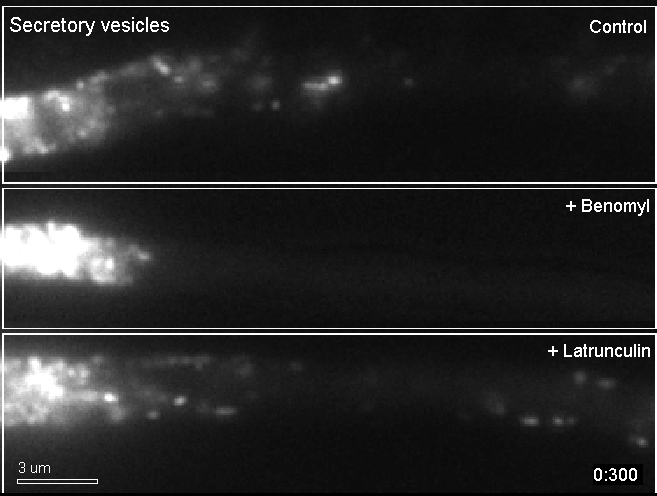

Supplement: Supplementary file 9 — Supplementary Movie 7 [file 41467_2025_59606_MOESM9_ESM.gif]

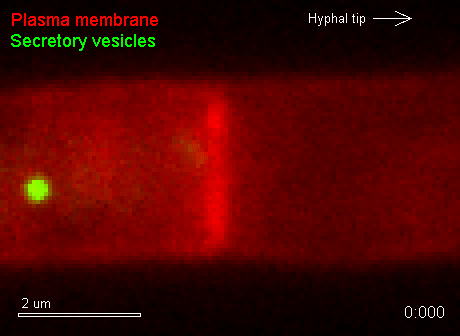

Supplement: Supplementary file 10 — Supplementary Movie 8 [file 41467_2025_59606_MOESM10_ESM.gif]

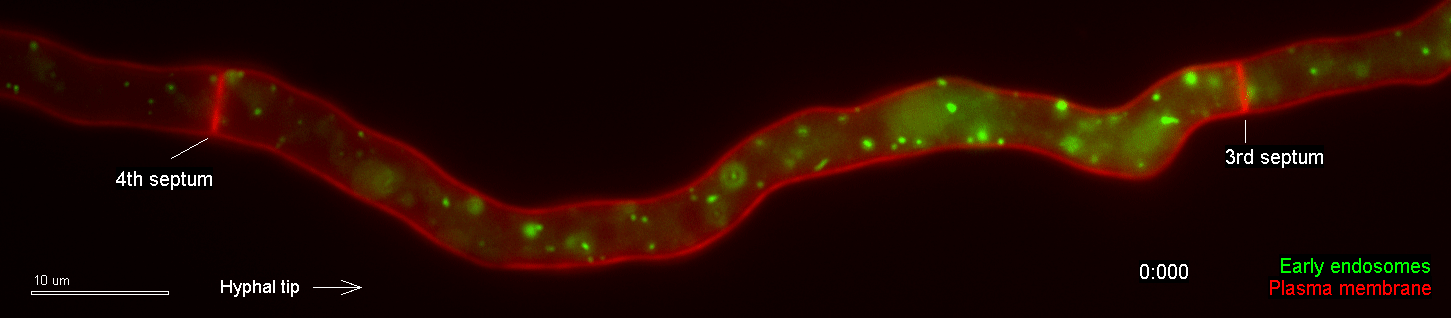

Supplement: Supplementary file 11 — Supplementary Movie 9 [file 41467_2025_59606_MOESM11_ESM.gif]

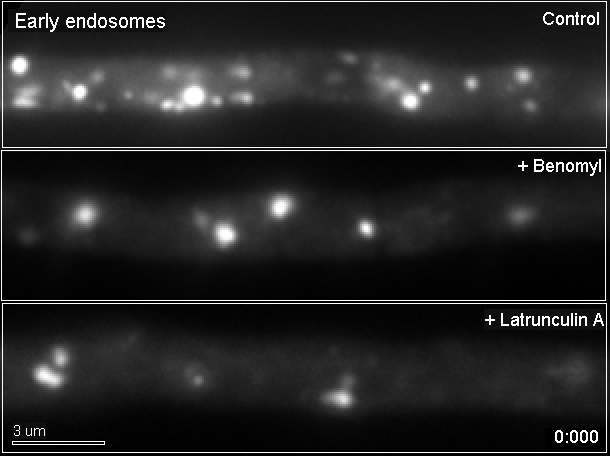

Supplement: Supplementary file 12 — Supplementary Movie 10 [file 41467_2025_59606_MOESM12_ESM.gif]

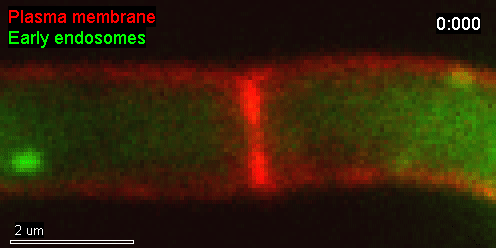

Supplement: Supplementary file 13 — Supplementary Movie 11 [file 41467_2025_59606_MOESM13_ESM.gif]

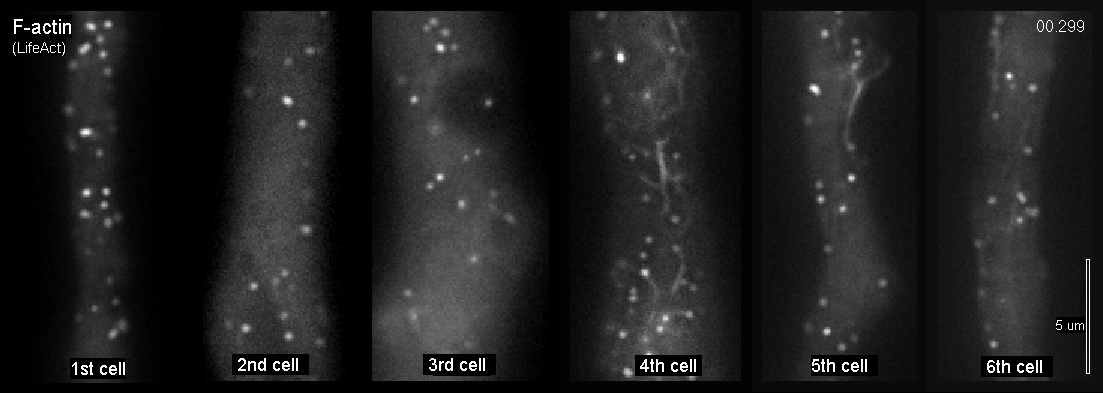

Supplement: Supplementary file 14 — Supplementary Movie 12 [file 41467_2025_59606_MOESM14_ESM.gif]

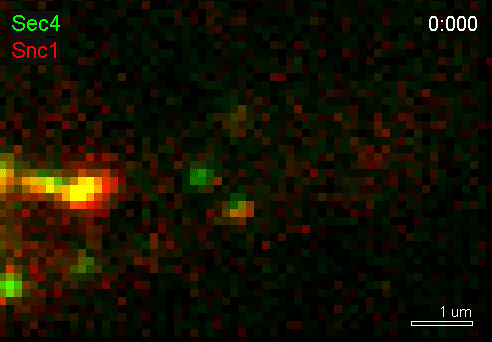

Supplement: Supplementary file 15 — Supplementary Movie 13 [file 41467_2025_59606_MOESM15_ESM.gif]

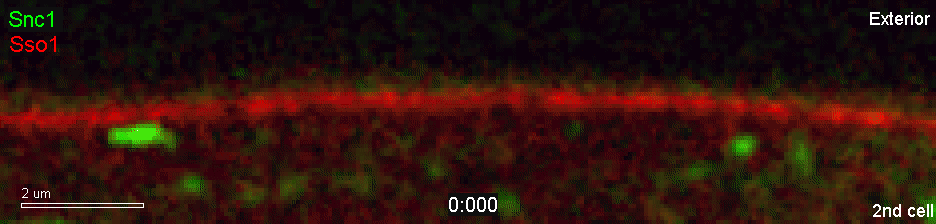

Supplement: Supplementary file 16 — Supplementary Movie 14 [file 41467_2025_59606_MOESM16_ESM.gif]

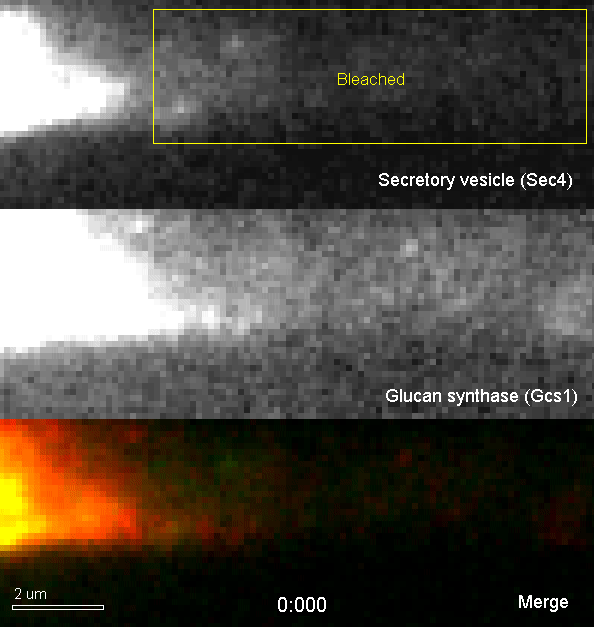

Supplement: Supplementary file 17 — Supplementary Movie 15 [file 41467_2025_59606_MOESM17_ESM.gif]

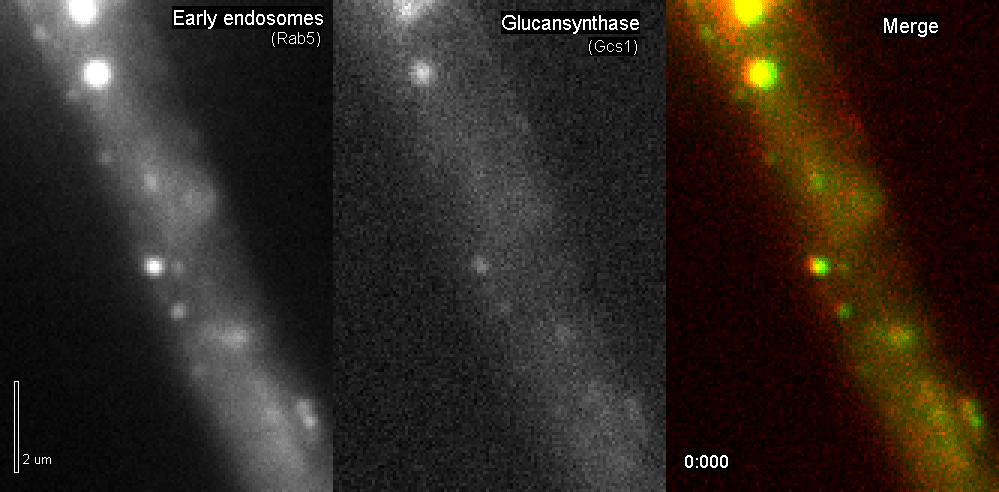

Supplement: Supplementary file 18 — Supplementary Movie 16 [file 41467_2025_59606_MOESM18_ESM.gif]

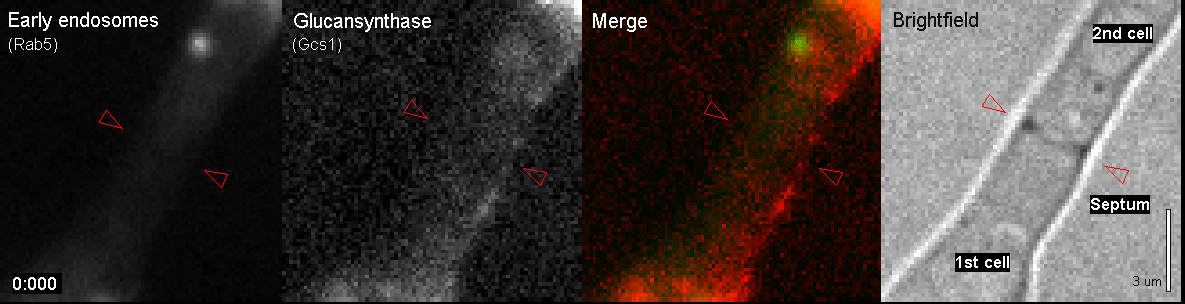

Supplement: Supplementary file 19 — Supplementary Movie 17 [file 41467_2025_59606_MOESM19_ESM.gif]
